# GK-1 effectively reduces angiogenesis and prevents T cell exhaustion in a breast cancer murine experimental model

**DOI:** 10.1007/s00262-023-03538-9

**Published:** 2023-09-22

**Authors:** Juan A. Hernández-Aceves, Jacquelynne Cervantes-Torres, Diana Torres-García, Francisco J. Zuñiga-Flores, Osiris J. Patiño-Chávez, Jorge A. Peña Agudelo, José Eduardo Aguayo-Flores, Yonathan Garfias, Laura Montero-León, Laura Romero-Romero, Armando Pérez-Torres, Gladis Fragoso, Edda Sciutto

**Affiliations:** 1 Departamento de Inmunología, Instituto de Investigaciones Biomédicas, Universidad Nacional Autónoma de México, Mexico City, Mexico; 2 Departamento de Biología Celular y Tisular, Facultad de Medicina, Universidad Nacional Autónoma de México, Mexico City, Mexico; 3 Unidad de Investigación, Conde de Valenciana, Instituto de Oftalmología, Mexico City, Mexico; 4 Departamento de Bioquímica, Facultad de Medicina, Universidad Nacional Autónoma de México, Ciudad Universitaria, Mexico City, Mexico; 5 Departamento de Patología, Facultad de Medicina Veterinaria y Zootecnia, Universidad Nacional Autónoma de México, Circuito Escolar, Ciudad Universitaria, Mexico City, Mexico

**Keywords:** 4T1-model, T cell exhaustion, Breast cancer, Angiogenesis, Immunotherapy, GK-1

## Abstract

Breast cancer is the leading malignancy in women worldwide, both in terms of incidence and mortality. Triple-negative breast cancer (TNBC) is the type with the worst clinical outcomes and with fewer therapeutic options than other types of breast cancer. GK-1 is a peptide that in the experimental model of the metastatic 4T1 breast cancer has demonstrated anti-tumor and anti-metastatic properties. Herein, GK-1 (5 mg/kg, i.v.) weekly administrated not only decreases tumor growth and the number of lung macro-metastases but also lung and lymph nodes micro-metastases. Histological analysis reveals that GK-1 reduced 57% of the intra-tumor vascular areas, diminished the leukemoid reaction's progression, and the spleens' weight and length. A significant reduction in VEGF-C, SDF-1, angiopoietin-2, and endothelin-1 angiogenic factors was induced. Moreover, GK-1 prevents T cell exhaustion in the tumor-infiltrating lymphocytes (TILs) decreasing PD-1 expression. It also increased IFN-γ and granzyme-B expression and the cytotoxic activity of CD8^+^ TILs cells against tumor cells. All these features were found to be associated with a better antitumor response and prognosis. Altogether, these results reinforce the potential of GK-1 to improve the clinical outcome of triple-negative breast cancer immunotherapy. Translation research is ongoing towards its evaluation in humans.

**Supplementary Information:**

The online version contains supplementary material available at 10.1007/s00262-023-03538-9.

## Introduction

Breast cancer is the leading cause of cancer-related death among women worldwide [1]. Triple-negative breast cancer (TNBC) is the most aggressive subtype, accounting for 10–20% of all breast cancer types. There are very only few therapeutic options [2, 3] and a very poor prognosis still prevails, Thus, the improvement of the treatment of TNBC is an urgent need [4].

Transplantable mouse mammary carcinoma 4T1 cells induce a deadly metastatic tumor that closely resembles human TNBC [5, 6]. This malignancy is accompanied by splenomegaly with splenic granulocytopoiesis and leukemoid reaction and granulocytosis. Indeed, 4T1 tumor cells secrete growth factors that can stimulate extramedullary myelopoiesis, and increased levels of Gr-1 (Ly6G) and CD11b^high^ myeloid-derived suppressor cells (MDSCs) [7]. These cells can inhibit the anti-tumor immunity, promoting tumor progression and metastatic niches [8, 9].

Breast cancer cells have developed various mechanisms for immune evasion, characterized by a reduction of its immunogenicity by different means, such as an increased expression of inhibitory receptors and an enrichment of the tumor microenvironment with T regulatory cells (Tregs), MDSCs, tumor-associated M2 macrophages, and T cell exhausted (Tex), among other cell types that promote tumor expansion and metastasis, worsening the patient condition [10]. Furthermore, tumor cells can induce their own blood supply from the preexisting vasculature in a process that mimics normal angiogenesis [11]. Currently, tumor angiogenesis is considered a critical target to treat breast cancer, as it is highly correlated with metastasis [12, 13]. Indeed, antiangiogenic therapy with bevacizumab, antibodies against vascular endothelial growth factor A (VEGF-A), or tyrosine kinase inhibitors such as sunitinib and pazopanib have been shown to effectively control metastatic breast cancer [14].

T cell exhaustion is associated with a prolonged exposure to antigen and/or inflammatory signals. Tex overexpress PD-1, CTLA-4, LAG-3, and TIM-3 among other inhibition receptors, and they are characterized by a progressive loss of effector functions, including an impaired production of effector cytokines and reduced proliferation and cytotoxic activity [10, 15]. Tex (PD-1^high^) can be used as a predictor of the responsiveness to immunotherapy with anti-PD-1/PDL-1 antibodies [16, 17]. However, the benefit of these immunotherapies is still controversial. While some studies show positive results, others are less encouraging [18–20]. Other approaches, such as use of recombinant IL-2 and IFN-γ [21, 22] or immunomodulatory molecules to activate the adaptive and innate immune responses have also been proposed to treat cancer [23, 24].

On the other hand, angiogenesis plays a central role in both local tumor growth and metastasis in breast cancer. Thus, anti-angiogenesis therapies for cancer have raised interest. However, only a low to moderate response in the outcome of TNBC patients has been observed. [25]

GK-1, an 18-aa immunomodulatory peptide originally identified in *Taenia crassiceps* and shared by other phylogenetically close cestodes, induces protection against cysticercosis [26]. It is possible that the adaptive immune response against cysticerci is mediated by MHC, since different motifs were theoretically predicted in the GK-1 sequence [27]. On the other hand, GK-1 promotes the activation of NFκB in macrophages and dendritic cells through MyD88 [28], which in turn promote an inflammatory environment that result in an anti-tumoral response.

Its anti-tumor properties were evaluated on murine experimental melanoma [29] and breast cancer [30]. In the latter, GK-1- immunotherapy reduced tumor growth, increased immune surveillance and dramatically reduced the number of macro-metastases. This study was designed to deepen on the anti-tumor and anti-metastatic properties of GK-1 in the murine model of mammary carcinoma 4T1.

## Material and methods

### Mice

Female BALB/c mice, aged 4–6 weeks, were obtained from the animal facilities at the Instituto de Investigaciones Biomédicas (IIB), Universidad Nacional Autónoma de México (UNAM). The animals were acclimated in the animal house and kept under controlled light (12 light /12 dark hours) and temperature (22–24 °C) conditions throughout the experiment. Food and water were allowed ad libitum.

### GK-1

GK-1 peptide (GYYYPSDPNTFYAPPYSA) was purchased from USV LTD, Mumbai Maharashtra, India (Lot No. RD0001). Mice were administered with 100  μL of a 1-mg/mL solution of the peptide in isotonic saline solution (ISS).

### 4T1 cell line

The 4T1 cell linepurchased from the American Type Culture Collection (ATCC, Manassas, VA), were grown in RPMI-1640 medium (Gibco, Thermo Fisher Scientific, Waltham, MA) supplemented with 10% serum fetal bovine (SFB, Gibco), and 1% penicillin and streptomycin (Gibco). Cells were maintained at 37 °C and 5% CO_2_ were detached with 0.05% trypsin/0.5 mM EDTA (Gibco).

### 4T1 implantation

4T1 cell viability was assessed by the trypan blue exclusion method [31], and 1000 cells/50 μL of sterile ISS were implanted subcutaneously into the lower mammary fat pad of each mouse. Mice were checked three times a week, monitoring tumor growth and the general condition of each animal. Tumors sized were measured as previously reported [30].

### GK-1 treatment

GK-1 was first i.v. administered once the tumor was 1 mm × 1 mm of size (Day 0), and then administrated then every 7 days for 28 days. (Supplementary Fig. 1).

### Lymphoid tissue and primary tumor processing

Spleen, primary tumors, and tumor-draining lymph nodes were collected on 7, 14, 21, and 28 days after first treatment (daft). The spleen and all primary tumors were weighed. For functional tests, the cells were extracted by macerating the tissues between two 70-µm membranes and, the suspension was passed through a 50-µm filter. Mononucleated cells were obtained from this suspension by density gradient with Ficoll-Plaque Plus®, following the manufacturer’s instructions and incubated with red blood lysis buffer for 5 min and washed. Cells were counted and used with > 90% of viability.

### Histopathology

After mice were euthanized with sevoflurane, lungs and regional lymph nodes were fixed by intratracheal perfusion with 700 µL of 10% buffered Zamboni solution at day 21 and 28. The tissues were dissected and kept in the same fixative at room temperature (RT) for 24 h. Histological Sects. (4-μm) were obtained and stained with hematoxylin & eosin (H&E). The slides were analyzed and photographed under a Nikon® DS-Ri1 and Zeiss® Axiocam 506 color light microscope. Metastatic foci were counted blindly in lymph nodes and in the five pulmonary lobes (four right, one left) at coronal and horizontal section planes, respectively. In the lungs, metastatic foci were classified as pleural/subpleural or parenchymal foci.

Spleens from both mice groups were used to demonstrate the erythrocyte pseudo-peroxidase activity, to define the limits between splenic cords (red pulp) and the periarteriolar lymphoid sheath (the white pulp, T- and B-cell compartment).

### Hematological evaluation

Blood (100 µL) was collected in EDTA-coated tubes on 7, 21, and 28 daft. Leukocyte numbers were obtained with an EXIGO automated veterinary hematology analyzer (Boule Medical A.B., Sweden). Differential leukocyte counts were blindly determined in methanol-fixed, Wright-stained peripheral blood smears under a light microscope (40X).

### Angiogenesis

Prior to euthanasia, mice were injected in the lateral tail vein with 25 μL of 2% (w/v) Evans blue dye (EBD) solution per gram of body weight to stain the vasculature, and then processed as described elsewhere [32]. Cryostat cutting was performed at − 15 °C to obtain 4-μm sections of primary tumors. Slide mounting and nucleus blue counterstain were performed with DAPI.

### Image analysis

Images were analyzed as described elsewhere, with slight modifications [33]. All images were adjusted by deblurring and deconvolution. Then, the software was trained to detect vessels using the Instellesis Trainable Segmentation program. Briefly, six random images per condition were chosen, and three classes were distinguished in those images: background, vascular region, and avascular region. A deep segmentation of 120 features was selected, and a suitable classification and identification of vascular regions were performed. Then, the images were uploaded, image analysis was performed, identifying each class. Vascular regions are expressed in pixels^2^ and µm^2^. Statistical analysis was performed with the software ZEN v.3.5 (Carl Zeiss) [33].

### Flow cytometry

The immunophenotype of spleen,  tumor-draining lymph node, and primary tumor cells was determined by flow cytometry. T lymphocytes were counted and phenotypes were described every 7 days until 28 daft. All assays were performed using samples with > 95% of viability, as measured by trypan blue exclusion. Effector (CD3^+^ CD44^+^ CD62L^−^) and regulatory (CD3^+^ CD4^+^ CD25^+^ FoxP3^+^) T lymphocytes, monocytes (CD11b^+^, Ly6C^high^), and granulocytic MDSCs (CD11b^+^, Ly6G^+^) were identified (Supplementary Table 1).

The cells were fixed with 4% paraformaldehyde for 20 min at 4 °C and permeabilized with the BD CytoFix/CytoPerm kit for intracellular staining and with eBioscience™ Foxp3/Transcription Factor Fixation/Permeabilization for nuclear staining. The cells were read in a NxT Attune cytometer with two lasers (red and blue) and seven reading channels. Data were analyzed with the software FlowJo vX 10.0v.

### Cytotoxicity assay

Spleen and tumor CD8^+^ T lymphocytes were purified by density gradient, and sorted with a FACS® ARIA cytometer, using a positive selection for CD3^+^ CD8^+^ (> 95% purity); cells were co-cultured with 4T1 cells stained with 1 µM calcein AM (Cat. No. C1430, Life Technologies, Carlsbad, CA) for 30 min. Tumor purified lymphocytes were cultured in a 1:1 ratio with tumor cells, while spleen lymphocytes were cultured in a 5:1 ratio. Cells were incubated for 4 h in RPMI-1640 plus 10% SFB at 37 °C and 5% CO_2._ Then, the cells were centrifuged, and the supernatant were analyzed by fluorimetry (excitation, 470 nm; emission, 535 nm). Fluorescence intensity was measured in 100 µL of supernatant for 5 min in a Synergy H1 Biotek® fluorimeter, and the mean fluorescence intensity (MFI) was recorded. Cytotoxicity rates were based on readings of maximum release controls (tumor cells stained and treated with 0.1% Triton X-100 for 45 min) and free release control (tumor cells only stained).

### Intracellular cytokines

The functional activity of T lymphocytes was evaluated with a non-specific stimulus, using the Leukocyte Activation Cocktail with BD GolgiPlug™ (PMA/Ionomycin + Brefeldin) using 1 μL of activator for every 10^6^ cells in 96-well plates. Cells were incubated at 37 °C under 5% CO_2_ for 6 h, then washed, fixed with BD Cytofix/Cytoperm™ for 45 min at 4 °C and read.

### Protein extracts from primary tumors

Primary tumors were immersed in liquid nitrogen and stored at − 80 °C. Proteins were extracted from 5 mg of tissue with 300 ml of lysis buffer containing protease inhibitors homogenized with an electric homogenizer (IKA, model T 10 Basic S1, fitted with dispersion tools S10N-5G, Cincinnati, OH) under constant agitation for 2 h at 4 °C. The homogenate was centrifuged for 20 min at 11,000 × *g* at 4 °C and protein concentration was determined by Lowry in the supernatant. Extracts were stored at –80 °C until used.

### Angiogenic factors

Angiogenic factors were quantified in tissue protein extract samples (*n* = 5 per group), randomly selected from three independent experiments. A commercial multiplex kit (Milliplex MAP Mouse Angiogenesis Growth Factor Magnetic Bead Panel, Cat. No. MAGPMAG-24 K, Merck Millipore, Burlington, MA) was used, and the samples were read in the Luminex Magpix (Xponent Software) system.

### Statistical analysis

Data were analyzed either by parametric or non-parametric tests, depending on the results of a Shapiro–Wilk normality test. Tumor progression, PD-1 expression, and cytotoxicity were compared by a two-tailed Mann Whitney U test. Area under the curve (AUC) values were compared with a Kruskal–Wallis’s test. Cytokine production was analyzed by MANOVA with Hotelling’s T-squared comparison. Differences were considered as statistically significant when *P* < 0.05* or < 0.01** or < 0.001***. All analyses were performed either in GraphPad Prism v.7.0 or R-Studio v.1.3.

## Results

### GK-1 reduced the number of micro-metastases in the pleura and in lung parenchyma

**Fig. 1:**
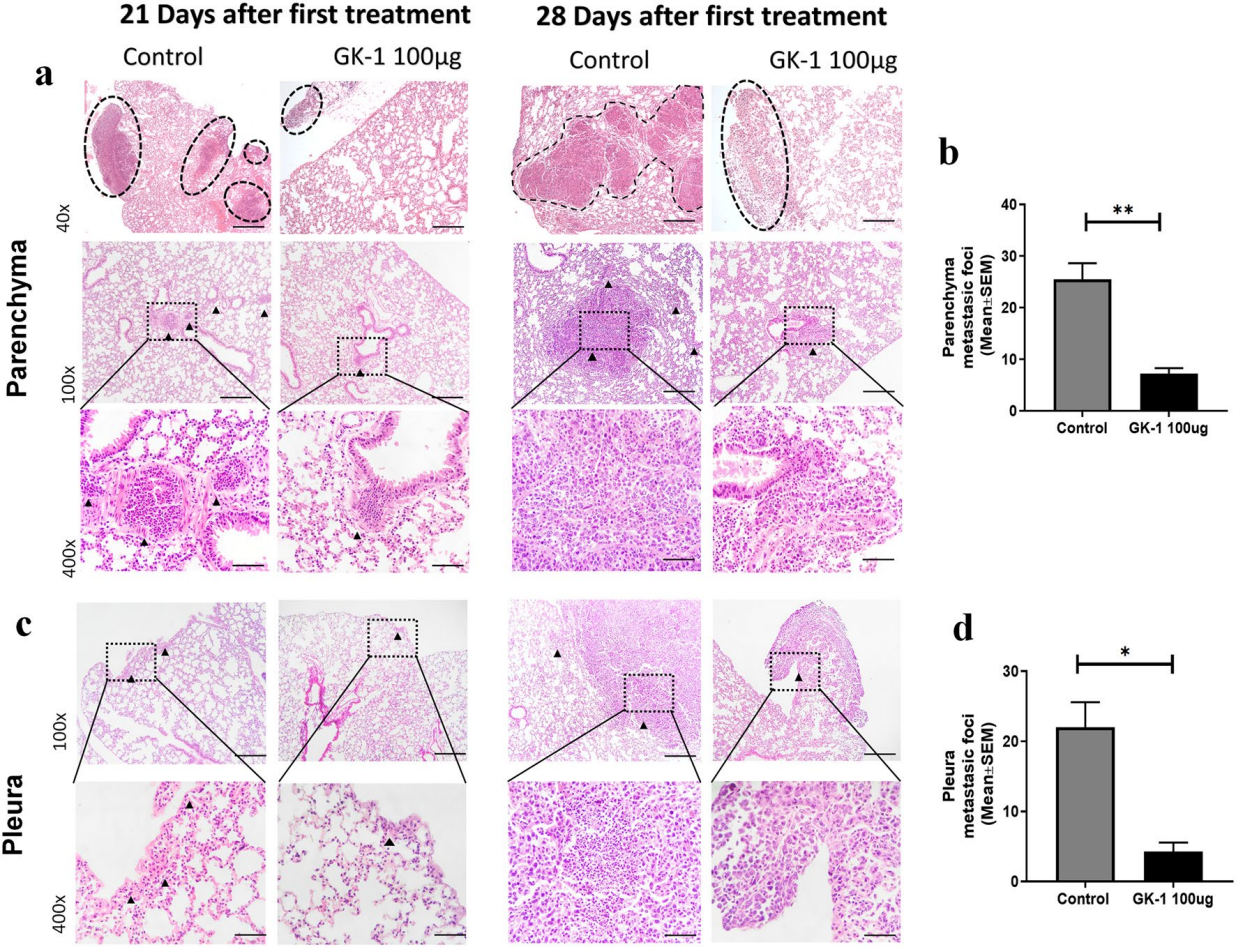
GK-1 decreased lung metastasis in the 4T1-induced breast cancer model. **a** Representative histological section of lung parenchyma and **c** pleura, stained with H&E on 21 and 28 daft. Treatment with GK-1 reduced metastatic focus dissemination until day 28. Metastatic foci are marked with black arrowheads. GK-1 significantly reduced the mean number of metastatic foci in lung parenchyma **b** and pleura **d** on day 28 after treatment. Metastatic foci were quantified considering a small group of cells with morphology that resembles 4T1 cells, showing nuclear abnormalities. Data are reported as mean ± SEM (n = 5 for each time). Significant differences in a two-tailed Mann–Whitney U test are marked as *P < 0.05 and **P < 0.01. Scale bar: 40x = 500 μm; 100x = 200 μm; and 400x = 50 μm

Previously, our research group reported that GK-1 decreased tumor growth rate in the murine model of breast cancer induced by the 4T1 cell line [30]. In line with those results, a significant decrease in tumor growth rate and weight were confirmed herein since 14 daft (Supplementary Fig. 2). GK-1 treatment reduced significantly the number and size of micro-metastatic foci in the lung, both the pleural/subpleural and parenchymal foci, in comparison with controls on 21 daft. This trend was even more evident on 28 daft (Fig. 1 a-d). Increased levels of infiltrated neutrophil in alveolar walls were observed more intensively in GK-1- than in control mice.

### GK-1 delayed metastases in tumor-draining lymph nodes (DLNs)

**Fig. 2:**
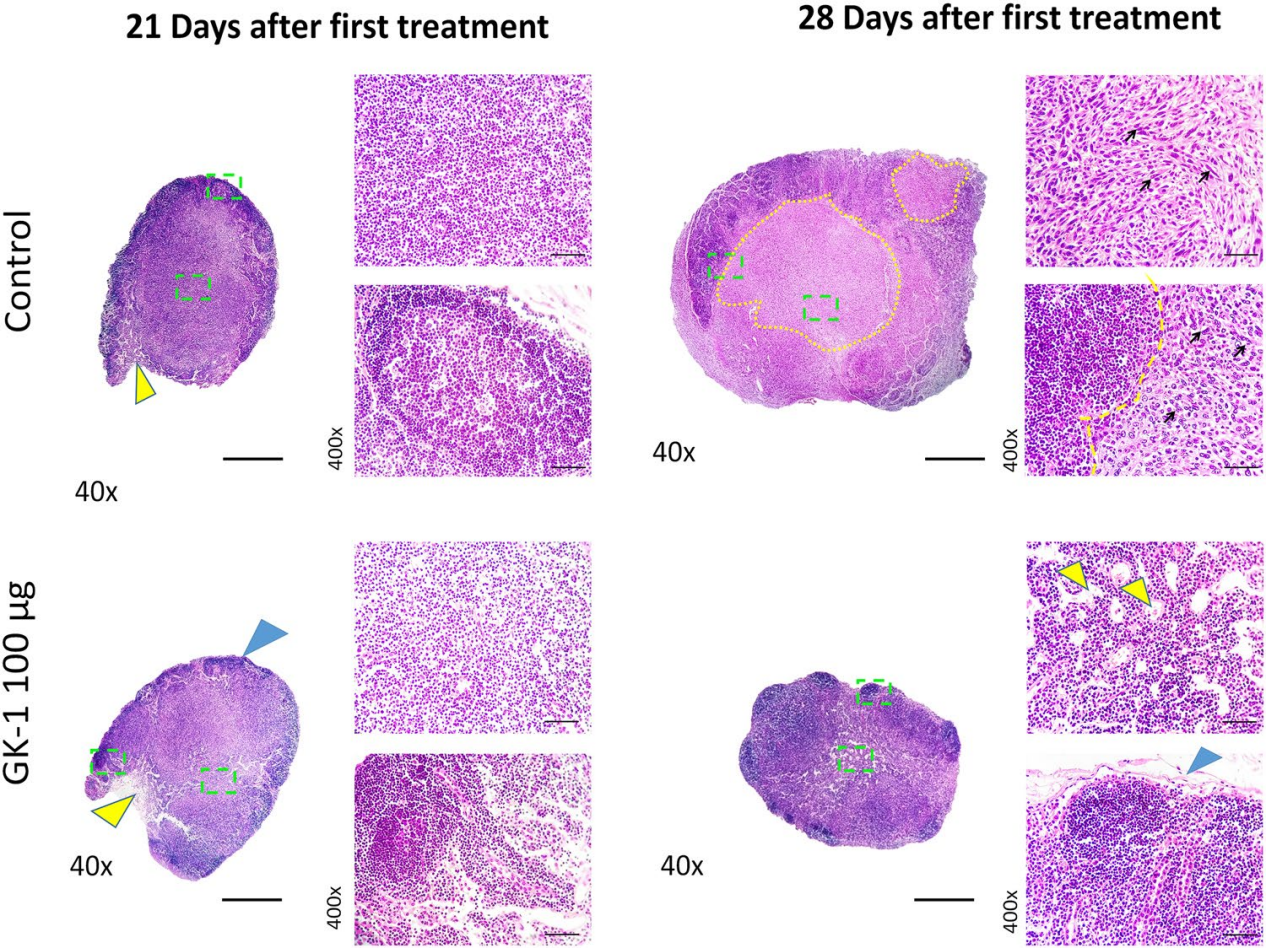
Representative histopathological sections of tumor-draining lymph nodes from ISS- and GK-1 treated mice in the breast cancer model induced by 4T1 cell line. In ISS-treated mice, blue and yellow arrowheads depict lymphatic sinus hyperplasia (subcapsular and medullar sinus, respectively). Paracortical and medullary lymphoid hyperplasia (central green dashed squares, and magnification) were also observed in the primary and secondary lymphoid nodules depicting polyhedral to spindle-shaped cells tumor cells with karyomegalic and pleomorphic nuclei (arrows indicate some of the tumor cells). Yellow dashed lines show increased size and metastatic eosinophilic foci in the inner cortex and medullary cords. In GK-1 treated mice, blue and yellow arrowhead showed hyperplasia in subcapsular and medullary sinuses, respectively, with conserved histological arrangement in the cortex, paracortex, and medullary cords (central green dashed square). Scale bar: 40x = 500 μm; 400x = 50 μm

On 21 daft, DLNs showed paracortical and medullar lymphatic sinus hyperplasia on histopathological examination, with no evidence of tumor metastases neither in GK-1- nor in ISS-treated mice (Fig. 2). On 28 daft, all tumor-draining lymph nodes examined in untreated mice (*n* = 4) showed increased size and extensive metastatic invasion, accompanied by eosinophilic foci; polyhedral to spindle-shaped tumor cells, with pleomorphic nuclei, were observed in the inner cortex and medullary cords; displaced cortical lymphoid tissue was distorted by peripheral metastases (Fig. 2). In contrast, no hyperplasia near subcapsular and medullary sinuses was observed in GK-1-treated mice, which showed a conserved histological arrangement in the cortex (with several primary lymphoid nodules), paracortex, and medullary cords (Fig. 2). The presence of tumor cells was detected in the medullary sinus in GK-1 treated mice, without the establishment of metastatic foci.

### GK-1 increased effector T lymphocytes and reduces intratumoral lymphocytes PD-1 expression

CD4^+^ and CD8^+^ effector T lymphocytes (CD3^+^/ CD44^+^/ CD62L^−^) were measured in spleen, DLNs, and tumor tissues, considering their relevance in the elimination of solid tumors and their value for tumor prognosis. Tregs were also quantified, considering their role in immunosuppression and tumor immune escape. The ratio of CD8^+^ T lymphocytes and Tregs is used as a biomarker for prognosis [34, 35].

**Fig. 3:**
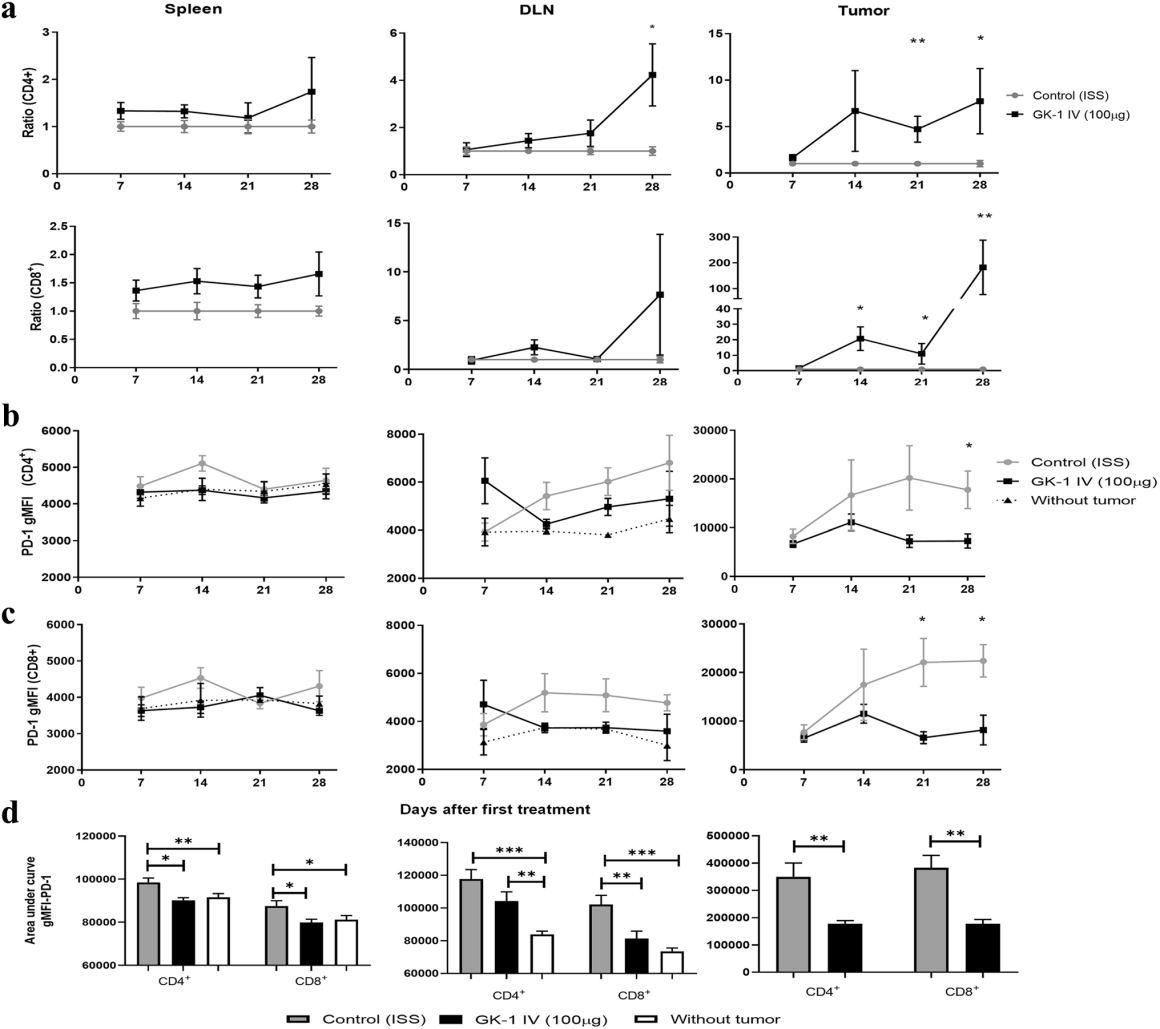
GK-1 increases effectors T lymphocytes in the tumor and reduces the PD-1 expression in effector T lymphocytes. **a** The control:control ratio is shown in gray lines, and the GK-1:control ratio is shown in black. A value > 1 indicates an increase with respect to control. PD-1 expression was measured by gMFI in effector T lymphocytes (CD3^+^ CD44^+^ CD62L^−^) from spleen, tumor-draining lymph node (DLN), and tumor tissues. PD-1 expression in CD4^+^ effector T cells on 28 daft. **b** in CD8^+^ effector T cells on 21 daft (**c**). Area under the curve for PD-1 gMFI on 28 daft (**d**). All data are expressed as the Mean ± SEM of 5 mice for each time and treatment. Two tails Mann Whitney U test was used. *P < 0.05 **P < 0.01, ***P < 0.001. The gating strategy is shown in Supplementary Fig. 4

As shown in Fig. 3a, only the intratumoral levels of CD4^+^ and CD8^+^ lymphocytes were significantly increased with respect to control mice after treatment with GK-1, a finding that implies that treatment with the peptide increased T cells infiltration, a marker associated with a good prognosis. The ratio CD8^+^ T lymphocytes/Treg was also increased in GK-1-treated mice (Supplementary Fig. 3).

### GK-1 decreased PD-1 expression in effector T lymphocytes

T cell exhaustion is characterized by an overexpression of inhibition receptors such as PD-1. The geometric mean fluorescence intensity (gMFI) of PD-1 was evaluated in effector T cells on 7, 14, 21, and 28 daft in spleen, tumor-draining lymph nodes and tumor tissues in GK-1-treated and untreated mice. As shown in Fig. 3b, GK-1 decreased PD-1 overexpression only in tumor CD4^+^ (28 daft) and CD8^+^ (21, 28 daft) T cells (Fig. 3b and 3c, respectively). The area under the curve (AUC) of PD-1 expression was lower in spleen, lymph node, and tumor tissues from GK-1-treated mice with respect to controls. Furthermore, similar levels of PD-1 expression were observed in tumor-free and GK-1-treated mice (Fig. 3d).

### GK-1 increased the cytotoxic activity and cytokine production

**Fig. 4:**
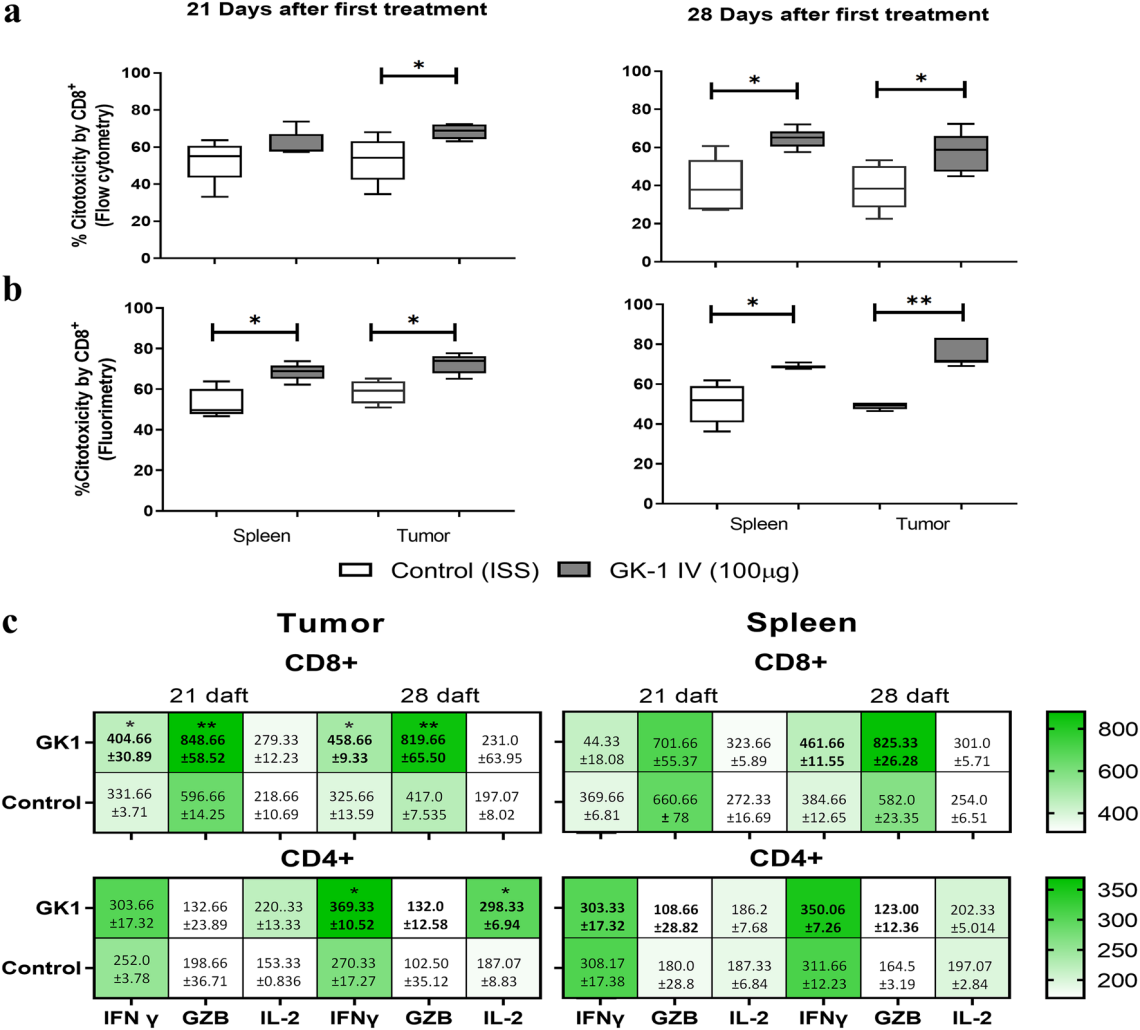
GK-1 treatment increased CD8 + cytotoxicity and the production of IFN-γ, GZB, and IL-2 by tumor-related T lymphocytes. **a** The cytotoxic activity of CD8^+^ cells was measured by their capacity to lyse 4T1 tumor cells previously labeled with fluorescent calcein-AM [% of cytotoxic activity = (4T1 without calcein/stained 4T1) × 100)]. Effector (CD8) to target (tumor cells) ratio was 5:1 in spleen and 1:1 in the tumor. **b** CD8 + cytotoxicity was estimated by measuring calcein release into the supernatant by fluorimetry. Data are reported as a boxplot with minimum and maximum values (*n* = 5 for each time and group). Differences were analyzed by a Mann–Whitney U test. **c** IFN-γ, GZB, and IL-2 production after stimulation with PMA/Ionomycin for 6 h. Heat map of gMFI and SEM from different intracellular products of T lymphocytes from tumor and spleen tissues (*n* = 3 for each time and group). Differences were analyzed by Hotelling’s T-squared MANOVA, *P < 0.05; **P < 0.01

Isolated CD8^+^ intra-tumoral and spleen T lymphocytes wereco-cultured with 4T1 cells. A higher cytotoxic activity, assessed by cytometry (Fig. 4a) and fluorimetry (Fig. 4b), was induced by intra-tumoral CD8^+^ T cells from GK-1-treated mice with respect to ISS-treated mice.

Since the effector activity of T cells is also affected by the intratumoral environment, the levels of IL-2, IFN-γ and granzyme B (GZB) were measured by flow cytometry in spleen and tumor tissues on 21 and 28 daft. As shown in Fig. 4c, GK-1 increased the levels of IL-2 and IFN-γ produced by tumor-related CD4^+^ and the levels of GZB and IFN-γ produced by tumor-related CD8^+^ T lymphocytes.

### GK-1 transiently altered the progression of leukemoid reaction

**Fig. 5:**
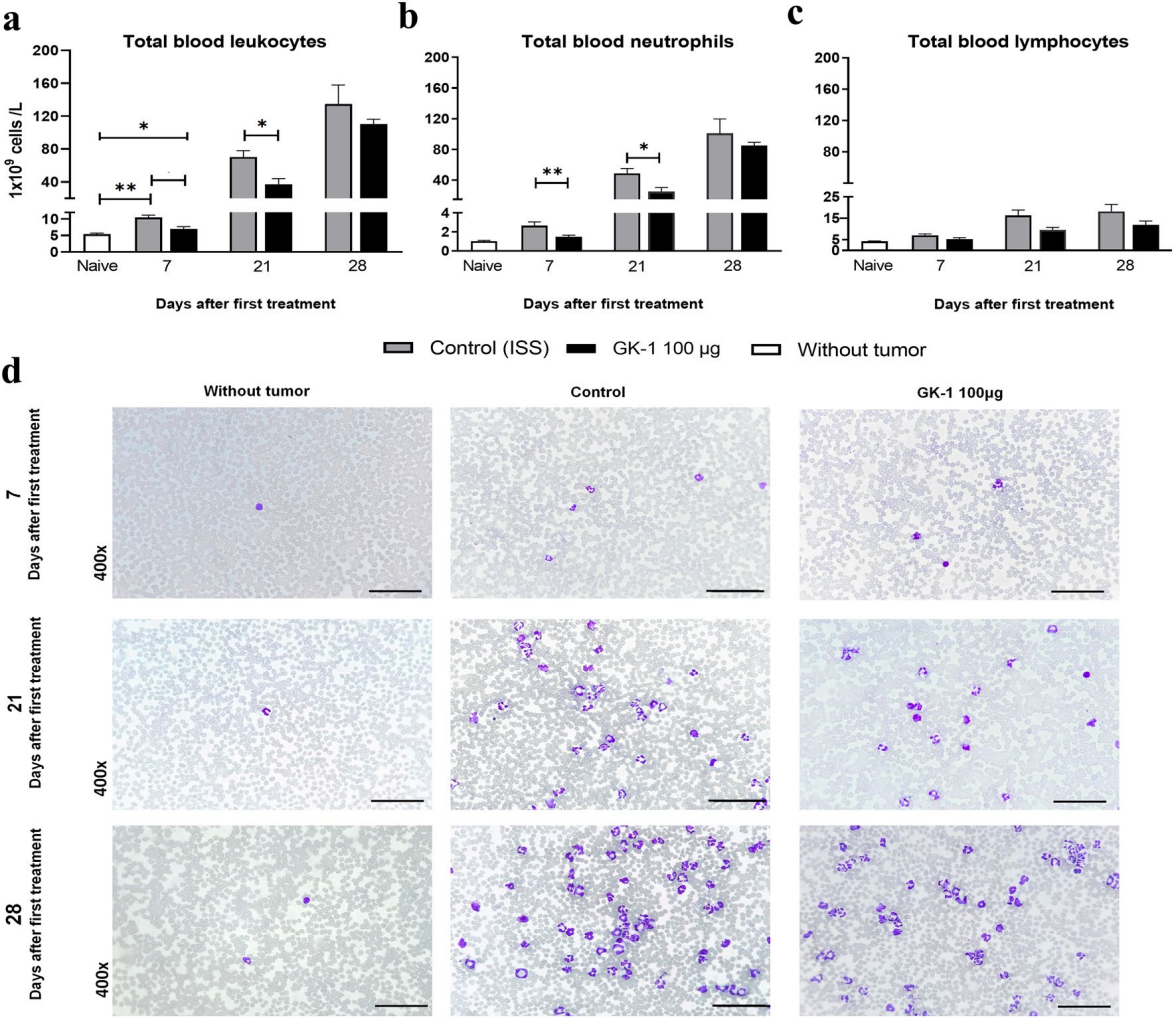
GK-1 modified the progression of the leukemoid reaction in the 4T1 cell-induced breast cancer model until day 21 of treatment. **a** Total blood leukocyte counts. **b** and **c** Total blood neutrophil and lymphocyte counts, respectively. Bars indicate standard error (SEM) (*n* = 5 for each time and group). Differences were analyzed by a two-tailed Mann–Whitney U test, *P < 0.05, **P < 0.01. Significant differences with respect to control were observed in all samples. **d** Representative images of blood smears from naïve, ISS-treated, and GK-1-treated mice on 7, 21, and 28 daft. Scale bars = 50 µm

4T1 breast cancer cells promote leukemoid reaction, a paraneoplastic effect related with tumor progression and metastasis, usually associated with a poor prognosis. It consists in a progressive leukocytosis and increased levels of immature granulocytes and monocytes like MDSCs [7]. As shown in Fig. 5, tumor growth was accompanied by an almost 25-fold increase in leucocyte levels with respect to the baseline of naïve mice, most notably a severe neutrophilia. In contrast, leukopenia with neutropenia were found on 7 and 21 daft of GK-1-treated animals with respect to ISS-treated mice (Fig. 5a and b). However, no statistically significant differences in leukocytosis were found between both groups on 28 daft.

No significant differences in lymphocyte counts between GK-1-treated mice and the ISS-treated group were found in the period under study, only between control animals and naïve mice on 7 daft (Fig. 5 c).

### GK-1 conserved spleen structure and reduced splenic myelopoiesis

**Fig. 6:**
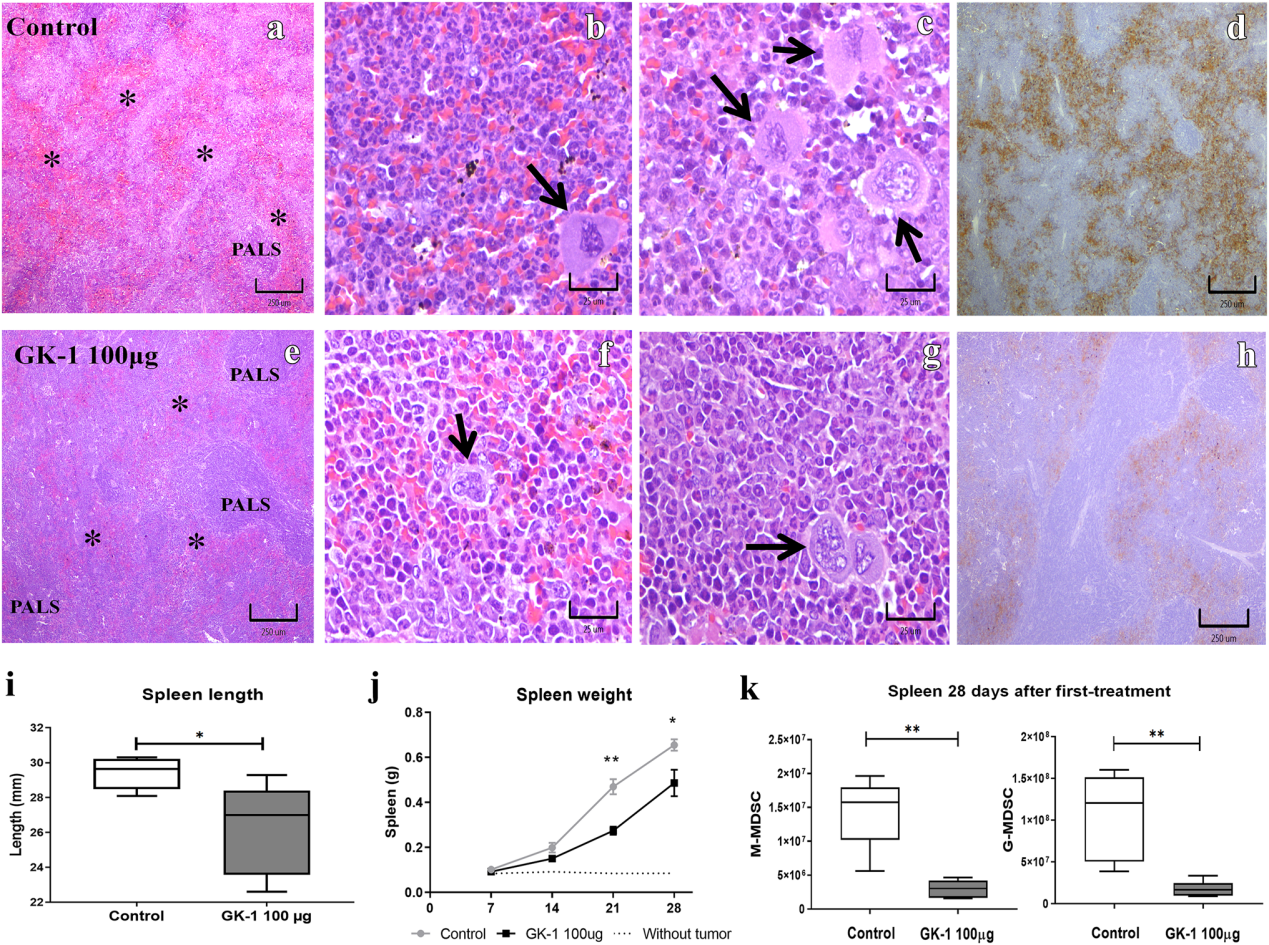
GK-1 preserved spleen structure and reduced splenic myelopoiesis. H&E stains of spleen slides. **a** Spleen in ISS-treated mice (control) show red pulp hyperplasia and a disorganized periarteriolar lymphoid sheath (PALS). **b**, **c** Numerous neutrophils, prominent megakaryoblasts (arrows), and macrophage-like cells were observed in the wide splenic cords of red pulp. **e** GK-1-treated mice showed a better-preserved histological organization of red pulp (*), with **f**, **g** lower counts and proportions of neutrophils, megakaryoblasts (arrows), and macrophage-like cells. Erythrocyte pseudoperoxidase activity at splenic cords **d** and **h** are shown in brown. **i** Mean ± SEM of spleen length. **j** Mean ± SEM of spleen weight in the three groups. **k** Absolute M-MDSC (CD11b^high^ Ly6C^+^) and G-MDSC (CD11b.^high^ Ly6G^+^ ) counts were decreased in GK-1 treated mice. Differences were analyzed by a two-tailed Mann–Whitney U test, *P < 0.05, **P < 0.01, (*n* = 5 per group)

Besides the leukemoid reaction, 4T1 tumor-bearing mice exhibit an extensive extramedullary hematopoiesis in the spleen, increasing its size [7] (Fig. 6).

As expected, both white and red pulp splenic compartments, showed clear histological alterations in untreated mice, evinced by changes in size. A diffuse increase in the amount of red pulp was observed, characterized by the presence of numerous leukocytes, mostly with neutrophil phenotype and macrophages, accompanied by numerous megakaryoblasts. The white pulp showed a significant decrease in cellularity, being difficult to identify the periarteriolar lymphatic sheaths in large areas of the spleen. In contrast, the spleens of mice treated with GK-1 showed a better distribution and proportion of white and red pulp. Increased levels of neutrophils, macrophages, and megakaryoblasts were also observed in the splenic cords (red pulp) of treated animals, but the numbers of these cell types showed different proportions, with a lower predominance of neutrophils. The erythrocyte pseudoperoxidase activity in the splenic cords helped to identify the extent and boundaries between the red and white pulp in spleens from both groups (Fig. 6 d,h).

Also, GK-1 significantly reduced the weight and length of the spleen with respect to ISS-treated mice (Fig. 6 i,j). This malignancy also triggers a myeloproliferative response, including MDSCs. Thus, the levels of granulocytic-myeloid derived suppressor cells (G-MDSC) CD11b^+^Ly6G^+^ Ly6C^low^ and monocytic- myeloid-derived suppressor cells (M-MDSC) CD11b^+^ Ly6C^+^[8] were measured in the spleen on 28 daft. As shown in Fig. 6k, a significant decrease of both suppressor cell types was observed after GK-1 treatment.

### GK-1 reduced angiogenesis in primary tumors

**Fig. 7:**
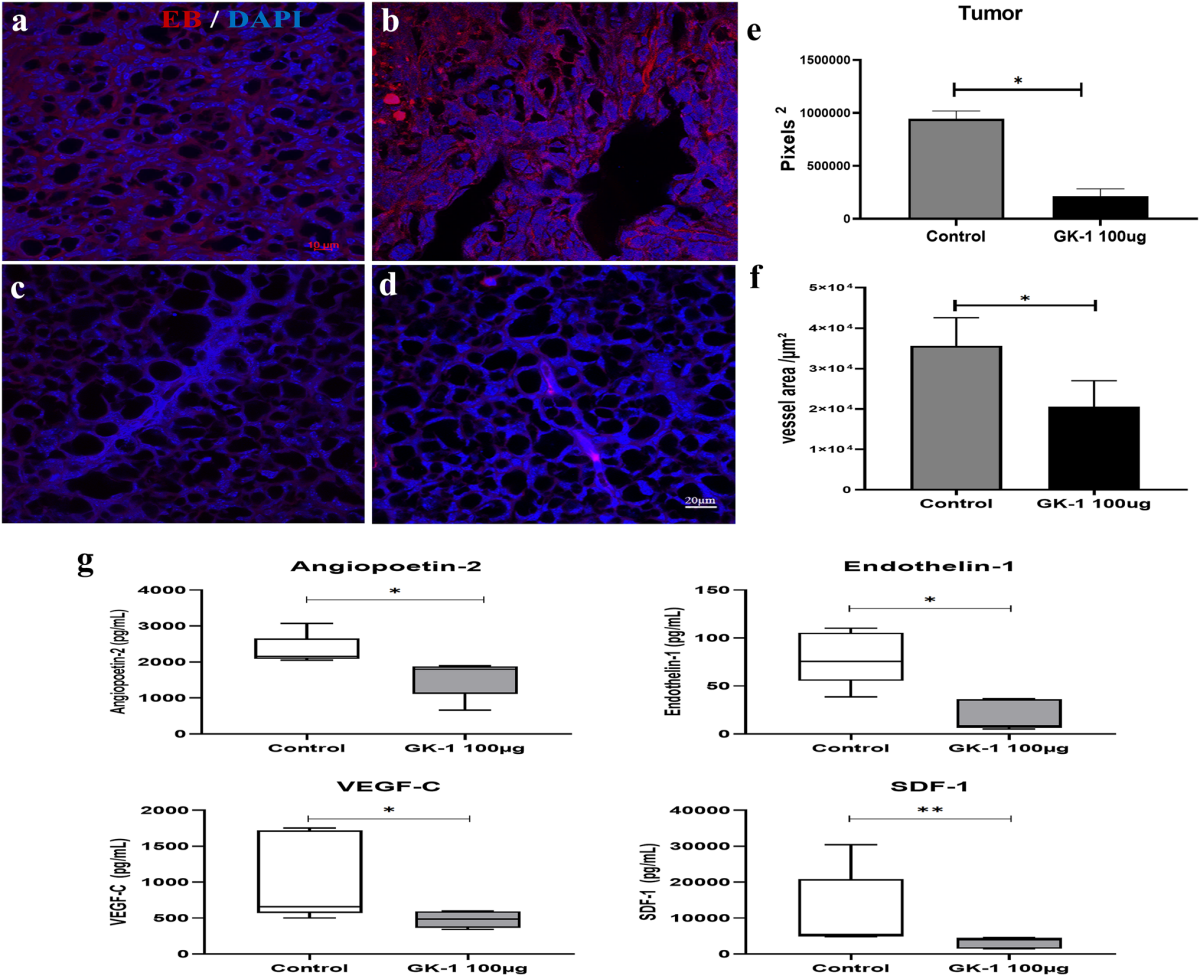
GK-1 reduces the blood vessel area, angiopoietin-2, endothelin-1, VEGF-C, and SDF-1 levels in tumors. **a**, **b** Two representative tissues sections of Evans blue (EB)-perfused primary tumors from ISS-treated, and **c**, **d** GK-1-treated mice counterstained with DAPI. **e**, **f** A measurement of pixels^2^ and vessel area (µm^2^) in randomly selected images with the Instellesis Trainable Segmentation software. The number of blood vessels inside tumor tissues from control animals was significantly higher than in GK-1-treated mice. **g** Angiogenic factors were quantified in protein extracts from tumor tissues with a Magpix® magnetic bead array (*n* = 5 per group). GK-1 treatment reduced the levels of angiogenic factors with respect to the control group. Data are reported as a boxplot, showing mean and 95% confidence levels. Error bars indicate standard deviation. Differences were analyzed by a two-tailed Mann–Whitney U test, *P < 0.05; **P < 0.01. Scale bar = 20 µm

Angiogenesis is a hallmark of cancer; as it is related both to metastasis and tumor growth [36]. Antiangiogenic therapy was been proposed to halt breast cancer progression [37]. As shown in Fig. 7 a, b, primary tumors in ISS-treated mice showed a more profuse vascularization than GK-1-treated mice on 28 daft (Fig. 7 c, d). This noticeable difference was further analyzed by evaluating EBD-perfused blood vessels in the primary tumor with the Instellesis Trainable Segmentation software. Blood vessel density (visualized by albumin-bound EBD) was significantly lower in GK-1-treated mice (Fig. 7 e, f, obtained with the software ZEN v.3.5, Carl Zeiss).

To corroborate the antiangiogenic effect in GK-1-treated mice, the level of expression of 13 angiogenic factors were measured. As shown in Fig. 7g, a significant reduction in the expression of angiopoietin-2, endothelin-1, VEGF-C, and SDF-1 was observed in GK-1-tretated mice, with respect to control or ISS-treated mice.

## Discussion

Breast cancer is the most prevalent and deadly malignancy among women worldwide; thus, finding novel therapeutic options and means of early detection is crucial to reduce the morbidity and mortality of the disease. The 4T1 cell line-induced breast cancer model is a highly metastatic and poorly immunogenic triple-negative tumors, useful for testing novel therapeutic approaches for TNBC [38, 39], especially for patients with both primary tumors and metastases [19, 20, 40]. We previously reported that GK-1 slowed 4T1 tumor growth and reduced the number of macrometastases [30].

In this study, we demonstrate that GK-1 immunotherapy dramatically decreased lung and lymph node micrometastases. Both effects are particularly relevant, since metastasis is responsible for about 90% of human deaths from breast cancer, [41] and lymph node infiltrating cells are markers of poor prognosis [42]. Moreover, GK-1 increased the number of intratumoral CD8^+^ T cells and its anti-4T1 cytotoxic and effector activity, evinced by the increased expression of IFNγ and granzyme in GK1-treated mice, all indicators of good prognosis in solid tumors [43]. GK-1 also increased the production of IFNγ and IL-2 in CD4^+^ T cells. These effects in the functional activity of T lymphocytes are accompanied by decreased levels of the inhibitory receptor PD-1 [44], an effect also observed. Altogether, these findings indicate that GK-1 could effectively enhance antitumor T cell immunity by promoting a robust, functionally active T cell infiltration into the breast tumor mass. The changes in tumor microenvironment induced by GK-1 could trigger an immunological conversion from a cold to a hot tumor, by remodeling the immunosuppressive microenvironment of 4T1tumors [45]. Cold tumors are characterized by a null or very low T cell infiltration, while hot tumors, with a better prognosis, have a high density of CD3^+^ and CD8^+^ T cells [46]. Thus, the conversion to hot tumors favored by GK-1 may be associated with a higher sensibility to immune checkpoint inhibitor therapies (ICITs) [47]. Furthermore, the effectiveness of ICITs such as anti-CTLA-4 and anti-PD-1/PD-L1 could represent a major improvement in life expectancy for patients with a variety of advanced cancer types [48]. However, as single agents, ICITs are only effective in a small subset of breast cancer patients [49]. Considering the results shown herein, it could be of interest to evaluate the therapeutic potential of GK-1 when co-administered with ICITs to improve the anti-tumor response. In this respect, promising results have been reported in a mouse model of melanoma [29].

Another result that merit comments is the capacity of GK-1 to decrease granulocytosis, leukemoid reaction, splenic myelopoiesis and megakaryocytes, factors related to splenomegaly [50, 51] and tumor progression. The decrease MDSCs—which induce pre-metastatic niches [52]— and red pulp hyperplasia could explain the reduction in spleen size and weight, both indicators of a reduction in extramedullary hematopoiesis, which in turn could be associated with the reduction in pulmonary metastasis. This reduction could be further favored by the decreased formation of new blood vessels in GK-1-treated mice, in clear contrast with the highly branched blood vessels in control mice, which promote a premature, abnormal angiogenesis [53].

Proangiogenic molecules, which could regulate endothelial cell proliferation and migration, were searched among 13 candidates in a multiplex assay. A significant decrease in the levels of angiopoietin 2, endothelin 1, SDF-1, and VEGF-C, all of them angiogenic factors involved in angiogenesis and vasculogenesis [11], was observed in GK-1-treated mice. VEGF-C, a growth factor of the vascular endothelial growth factor family receptors, endothelin 1, and SDF-1 exhibit a high pro-angiogenic activity by promoting mitosis and inhibiting apoptosis on endothelial cells, resulting in an increased vascular permeability and a promotion of cell migration, favoring tumor angiogenesis. In addition, VEGF promotes the recruitment and proliferation of immunosuppressive cells like Treg cells and MDSCs [54]. Thus, the reduction in the counts of these two suppressor cells in GK-1-treated mice could be partly mediated by the decrease in VEGF levels.

It is important to mention that angiopoietin 2 is one of the best characterized factors of an important family of proteins that promote a weakening of newly formed blood vessels branches. High concentrations of these factors have been reported to correlate with a poor patient prognosis [55, 56]. Interestingly, IFN-γ secreted by CD8^+^ CTLs has been linked to a suppression of tumor angiogenesis by reprogramming tumor-associated macrophages from an M2- to an M1-like type [57]. Thus, the presence of IFN-γ^+^ CD8^+^ T lymphocytes in GK1-treated mice may be contributing to control tumor-promoted vascular remodeling.

Overall, these evidences indicate that GK-1 could decrease tumor growth by activating intra-tumoral T cell immunity, whilst inhibiting angiogenesis and extra-medullar myelopoiesis, exerting both immune and vascular functions, which currently are two major targets to improve the prognosis in TNBC cases. These properties make GK-1 a potential next-generation therapeutic agent against the highly mortal TNBC.

### Supplementary Information

Below is the link to the electronic supplementary material.

Supplementary file1 (TIF 1553 kb)

Supplementary file2 (TIF 62609 kb)

Supplementary file3 (TIF 64561 kb)

Supplementary file4 (TIF 63768 kb)

Supplementary file5 (TIF 66975 kb)

## Data Availability

The datasets generated during and/or analyzed during the current study are available from the corresponding author on reasonable request.
